# Sensitization of avian pathogenic *Escherichia coli* to amoxicillin *in vitro* and *in vivo* in the presence of surfactin

**DOI:** 10.1371/journal.pone.0222413

**Published:** 2019-09-12

**Authors:** Jiaxu Liu, Xu Wang, Weiqi Shi, Zhuoyu Qian, Yongqiang Wang

**Affiliations:** 1 Haid Central Research Institute, Animal Husbandry and Fisheries Research Center of Guangdong Haid Group Co., Ltd, Guangzhou, China; 2 Guangzhou Foreign Languages School, Guangzhou, China; USDA-Agricultural Research Service, UNITED STATES

## Abstract

The purpose of this study is to assess the antibiotics adjuvant effect of surfactin for boosting the treatment effect of amoxicillin. Surfactin is used as a surfactant to mediate flux of mono-and divalent cations, such as calcium, across lipid bilayer membranes. In this study, we demonstrated that surfactin can increase the activity of amoxicillin against avian pathogenic Escherichia coli (APEC) in vitro with antimicrobial assays such as minimum inhibitory concentrations (MIC) and fractional inhibitory concentration (FIC). Additionally in the model of chick infection, surfactin exerted adjuvant effects with amoxicillin against APEC by lowering the numerical value of mortality and liver bacterial loads, and regulating the expression of inflammatory cytokines et al. We concluded that surfactin can act as a novel antimicrobial adjuvant with amoxicillin against AEPC infection in chicken.

## Introduction

Poultry is one of the most important meat resources all over the world. In order to promote the growth of poultry, a large number of antimicrobials had been used to prevent and treat diseases when raising poultry flocks[1]. It was well-known that the overuse of antibiotics can cause the emergence of antimicrobial resistant pathogens, which lead to the failure of treatment as well as be the resource of resistant bacteria/ genes that may threaten human health[2].

Avian pathogenic *Escherichia coli* (APEC), a common bacterium that is the principal cause of morbidity and mortality in poultry[3], and the antimicrobial resistance of APEC keeps increasing in recent years for higher incidence of resistant APEC microorganisms and higher MICs (Minimum Inhibitory Concentrations). Amoxicillin has been used in APEC infection as an effective treatment, however, the incidence of amoxicillin resistant of APEC strains reached up to 74–100% among 83 diseased chickens in Jordan[4], Brazil[5] and Thailand [6]from 1999 to 2014. Therefore, new effective therapeutic options for treatment of APEC infections are urgently needed. The use of antimicrobial adjuvants is an attractive strategy to address the issue of antimicrobial resistance[7]. The adjuvants may not have significant antibiotic activity itself during treatment, however, would improve the biologic activity of antibiotics when used in the combination[8].

In the research, we take advantage of the characteristic of surfactin as antimicrobial adjuvants. Surfactin is a cyclic lipopeptide biosurfactant, which is the potential replacement for synthetic surfactants in food, biomedical and pharmaceutical industry[9]. The amphiphilic nature of surfactin allows its easy incorporation nano-formulations, such as polymeric nanoparticles, micelles, microemulsions, liposomes[10]. The properties of nano-formulations offers surfactin as an agent to improve antimicrobial ability[11].

We demonstrated the bactericidal effect of surfactin and amoxicillin by minimum inhibitory concentrations (MIC) and fractional inhibitory concentration (FIC) assays *in vitro*, and the prognosis of chicks that challenged with APEC after treatment are shown by mortality rate, histopathological changes and bacterial loads. In additional, we also checked the expression levels of inflammation related cytokines at the end of experiment to evaluate the animals’ health condition. These results highlight the potential of surfactin as a novel antimicrobial adjuvant to handle infections.

## Materials and methods

### Bacterial strains

Avian pathogenic *Escherichia coli* (APEC) O78 strain was obtained from the preservation of Haid institute at Guangdong province, China, and the strain was prepared in LB medium. Surfactin was purchased from Department of Chemistry, Zhejiang Univ, China.

### Minimum inhibitory concentrations (MIC) of surfactin and amoxicillin

The MIC of surfactin /amoxicillin was determined by broth microdilution method in 96-well microtiter plate[12]. Prior to commence two-fold serial dilutions of surfactin/ amoxicillin in the MH media, surfactin and amoxicillin were dissolved in distilled water at 1000 μg/ml and 2000 μg/ml, respectively. After adding the *E*. *coli* (1×10^6^ colony forming units [CFU]/mL) into each well with surfactin/ amoxicillin at two-fold serial dilutions in same volume, the mixture was incubated at 37°C for 24 h. The MIC was recorded when the lowest concentration of surfactin inhibited the growth of test bacterial (optically clear). The controls were prepared using MH and APEC O78 strain, respectively.

### Synergistic effects of surfactin on *E*.*coli*

The antibacterial effects of surfactin in combination with amoxicillin was assessed using the checkerboard test[13]. Fractional inhibitory concentration (FIC) index were calculated using the following formula: FIC = (MIC drug A in combination/MIC drug A alone) + (MIC drug B in combination/MIC drug B alone). Briefly, surfactin was serially diluted in MH and each dilution (50 μl) was added into 96-well plate. Prior to adding bacteria to the wells, amoxicillin was serially diluted and 50 μl was dispensed in each well. Then, an overnight culture of APEC O78 strain was adjusted to 1×10^6^ CFU/mL, and 100 μl of them were added to each well. After 16 h of incubation at 37°C, the FIC was recorded same as MIC described above.

FIC index is indicated as synergy at values of less than 0.5, partial synergy at values greater than 0.5 and less than 1, an additive effect for values of 1, indifferent effect for values greater than 1 and less than 4, and an antagonistic effect for values of 4.0 or greater[14].

### Animal experiment

Chicks and the procedures used for this study were following a standard protocol reviewed and approved by Institutional Animal Care and Use Committee of Jilin University (approval no JLU-20150226), following the strict compliance with requirements of the Animal Ethics Procedures and Guidelines of People’s Republic of China. 105 1-day-old partridge chicks (Foshan Xinguangmu Agriculture and Animal Husbandry Co., Ltd, Guangdong, China) inoculated with Marek’s disease vaccine were randomly assigned into 7 groups (15 chicks/group) as shown in Table 1. All birds were fed with antibiotic free feed during the experiment. At day-5, All groups except the control were challenged with 0.2 ml of 3×10^8^ CFU APEC O78 strain through the subcutaneous injection of neck. And the amount of injection was based on the body weight of chicken at experiment day 5. All chickens from group 1 to 5 except group 6 were treated three times a day during 6 days’ treatment period after *E*.*coli* challenge. Surfactin and amoxicillin were given orally after dissolved in water. The duration of the experiment was 9 days, all chicks had free access to food and water and were kept in a temperature-controlled room (32±0.5°C). In order to reduce the chicks suffering, the chicks’ clinical symptom was used to determine when chicks should be euthanized by cervical vertebra dislocation, such as: hunched, lethargic, reluctant moving and dyspneic, during the 6 days’ treatment period after *E*.*coli* challenge and prognosis period (3 days after treatment period). The Mortality and clinicopathological changes were recorded 3 times every day after inoculation for shorten the time of chicks suffering furtherly. In addition, pathological changes were recorded using colibacillosis lesions method as described previously [15]in air sac, lung, liver and pericardium in Table 2, and clinical signs of chicks were monitored as the description of Andreas[16]. After prognosis period, all the chicks were weighed and euthanized and directly submitted to post mortem analysis.

**Table 1:** Overview of experiment groups.

Group	Treatment	Daily dose/g bodyweight, 3 times per day
1	Amoxicillin	0.02 mg/g^1^
2	Amoxicillin	0.01 mg/g^1^
3	Amoxicillin and surfactin	0.01 mg/g amoxicillin^1^ + 0.01 mg/g surfactin
4	Amoxicillin and surfactin	0.005 mg/g amoxicillin^1^ + 0.005 mg/surfactin
5	Surfactin	0.01 mg/g
6	Positive control	none
7	No challenged	none

^1^Corresponding to 20 mg Amoxicillin/kg bodyweight.

**Table 2:** Colibacillosis lesion scoring.

Tissue	Score	Lesions
Air sac	0	No lesions
	1	Mild cloudiness and/or pinhead-size foci of fibrinous exudate
	2	Very cloudy and/or widespread presence of fibrinous exudate
liver	0	No lesions
	1	Small amount of fibrinous exudate over hepatic surface
	2	Large amount of fibrinous exudate over hepatic surface
pericardium	0	No lesions
	1	Small amount of fibrinous exudate over pericardium surface
	2	large amount of fibrinous exudate over pericardium surface

### Histopathology

After prognosis period (3 days after treatment period), organs including liver, lung, spleen, thymus, bursa of fabricius and small intestine from chicks of per group were collected and fixed with 4% paraformaldehyde. The fixed tissues were processed routinely for histopathological examination following the standard method[17].

### Bacterial load in liver

The right side of liver from all the chicks in this experiment were dissected and ground, and the particles were serially diluted in PBS. And 100 μl of each dilution was inoculated on Maconkey agar APEC selective plates. After 16 hours incubation at 37°C, the amount of CFU/g tissue was determined by counting the bacterial colonies.

### Cytokine expression analysis by relative real time Q-RT-PCR

The total RNA of liver sample from euthanized chicks after prognosis period was extracted using TRIZOL reagent (Invitrogen, USA), and cDNA was synthesized using random primers. The primers of inflammatory cytokine IL-1β, TNF-α, IL-10 and IL-13 were designed as shown in Table 3. Each real-time PCR reaction (10μl volume) contained 5 μl SYBR Green real-time PCR master mix (TAKARA), 0.25 μM gene-specific primers, and 1μl standardized template cDNA. Amplification conditions included as follows: 95°C for 10 min, followed by 39 cycles of 95°C for 15 s, 60°C for 1 min. Each relative expression was calculated as the ratio of inflammatory cytokine to the GAPDH gene using 2^-ΔΔCT^ method.

**Table 3:** The primer of chicks’ cytokine.

RNA target	Primer sequences (5'-3')	Size for PCR product (bp)	Accession no.
IL-1β	F: GGGACTTTGCTGACAGCGACCTG R: GTCGAAGGACTGTGAGCGGGTGT	128	Y15006
TNF-α	F: GCCCTTCCTGTAACCAGATG R: ACACGACAGCCAAGTCAACG	71	HQ739087
IL-10	F: CGGGAGCTGAGGGTGAA R: GTGAAGAAGCGGTGACAGC	272	AJ621614
1L-13	F: CCAGGGCATCCAGAAGC R: CAGTGCCGGCAAGAAGTT	256	AJ621735
GAPDH	F: gagaaaccagccaagtatgatga R: cttgacgaaatggtcattcagt	180	K01458

### Statistical analysis

Data were expressed as the mean ± standard deviation (SD). Differences between groups were analyzed by ANOVA using SPSS. The differences were considered significant at P<0.05.

## Results

### Synergy assessment *in vitro*

For APEC O78 strain, the MIC of amoxicillin and surfactin were 1000 ug/ml and >1mg/ml, respectively. The FIC index between amoxicillin and surfactin was 4>FICI>1, as an indifferent effect *in vitro*.

### Mortality and clinicopathological changes

**Fig 1:**
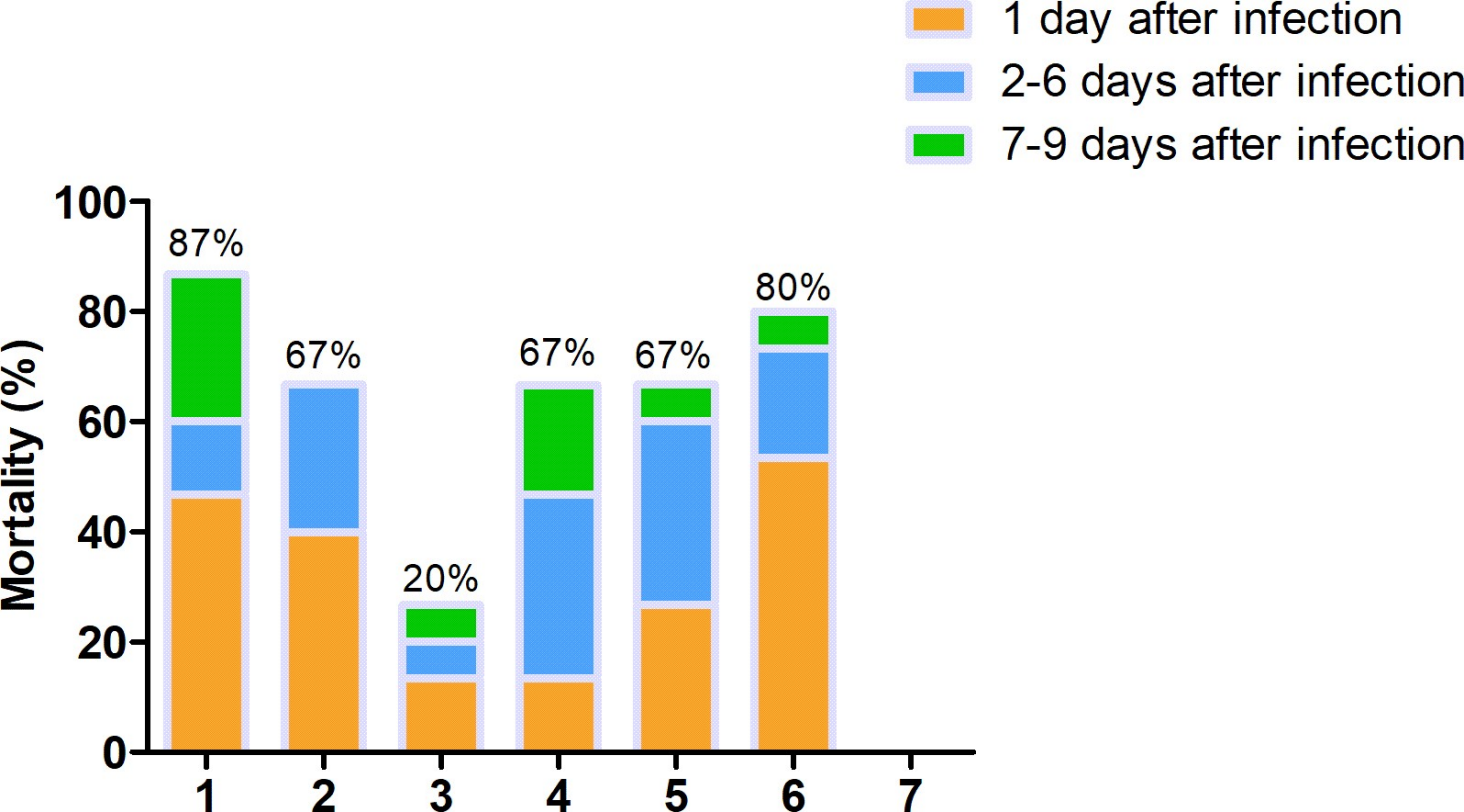
The mortality of dead chicks and euthanized chicks during the experiment. In 1 day post inoculation, called acute dead period, the mortality of group 6 is 42% as the highest, and mortality in the group 3 is 17% as the lowest. In the 2–6 days post inoculation, called chronic dead period, the mortality of group4 is higher than other groups, and the data of group 3 is the lowest. In the 7–9 days, called prognosis period, the mortality of group 1 is higher than others, and there is no dead chicks’ appearance in group 3.

After inoculation of *E*.*coli*, the chicks were observed every 8 h until the end of the experiment. Chicks in the control group remained healthy throughout the experiment period without any clinical signs. The mortality of chicks in each group was shown in Fig 1 at first nine days. It showed different mortality in acute dead period after day 1 following *E*.*coli* challenge, and a severe depression in all groups in this period except the blank group. All chicks displayed abnormal avian behavior, including hunched, lethargic, reluctant moving and dyspneic. With the ongoing of treatment, the status of chicks from group 3 to 5 improved slightly. Comparing the mortality between group 1 and 3, it showed that surfactin in combination with amoxicillin significantly decreased the mortality from 87% to 20%, and reducing the dosage of amoxicillin. Interestingly, it could be seen from the data in Fig 1 that there was no death in group 3 at prognosis period, and the mortality of group 3 was the lowest among the experiment groups.

**Fig 2:**
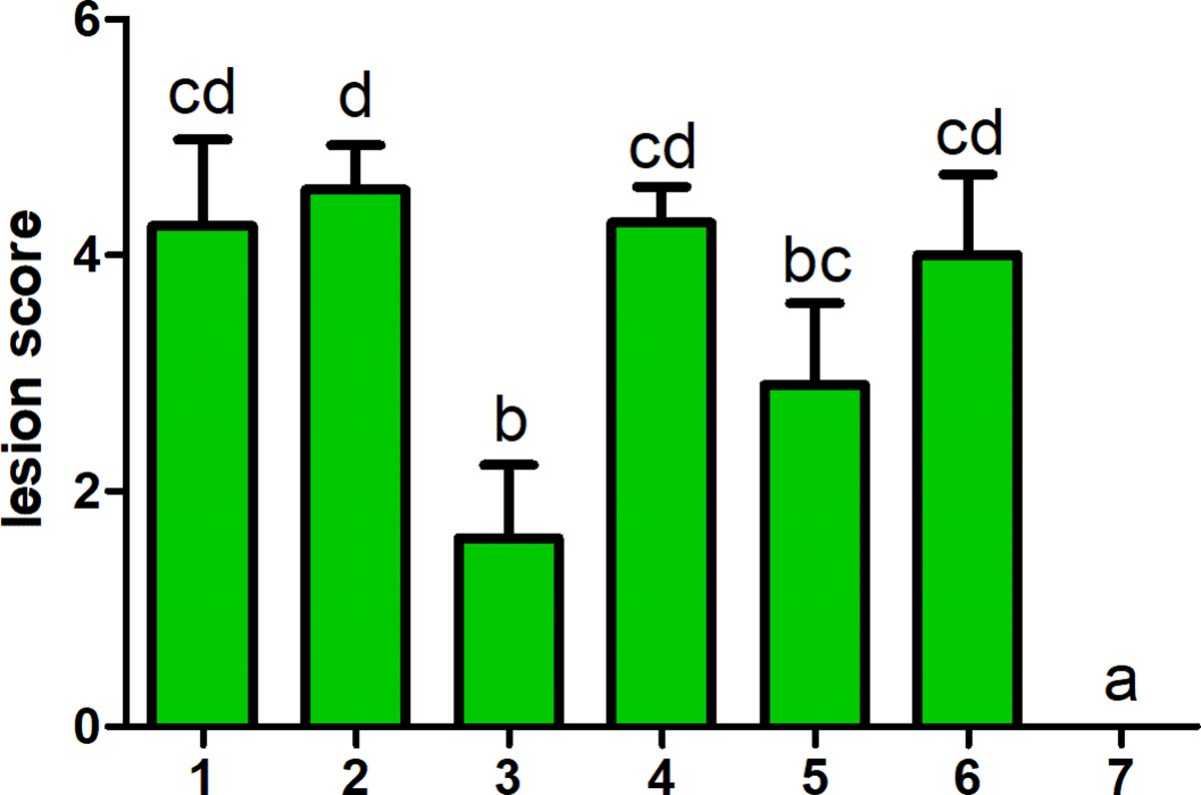
Score of pathology lesion following dead chicks and euthanized chicks after acute dead period. Significant difference between group 3 and other groups is found. Groups with different letters are significantly different (P < 0.05).

In order to furtherly evaluate the effects of amoxicillin and surfactin, the deceased chicks in the experiment were necropsied. There were no significant pathological changes of APEC infection in all groups at the acute death period, however, there were significant APEC infection pathological changes in the chronic death period and prognosis period except the unmedicated control group, such as aspericarditis, perihepatitis and airsacculitis. After acute death period, the colibacillosis lesion scores of dead chicks were recorded as shown in Fig 2. Comparing the colibacillosis lesion scores among all treatment groups, the score of group 3 was lower than other groups (P<0.05).

### Bacterial load in liver

**Fig 3:**
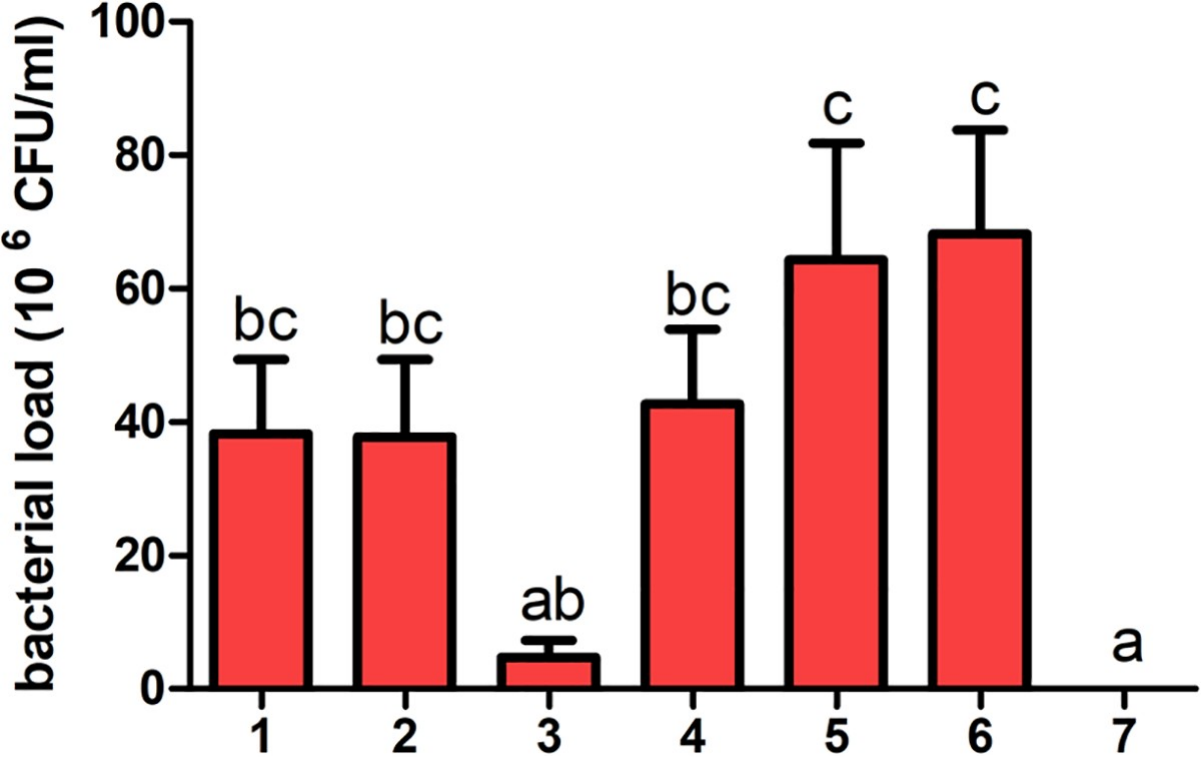
Bacterial load of dead chicks and euthanized chicks’ liver during the experiment. During the experiment, Significant difference of liver bacterial load between group3 and other groups is observed. The groups with different letters are significantly different (P < 0.05).

APEC loads in the liver of dead and euthanized chicks was measured after the prognosis, and the result was shown in Fig 3. The bacterial loads of chicks showed that the obvious APEC infection pathological changes score was different, and the data from group 3 was significantly lower than positive control and group 5 (P<0.05).

### Histopathology

**Fig 4:**
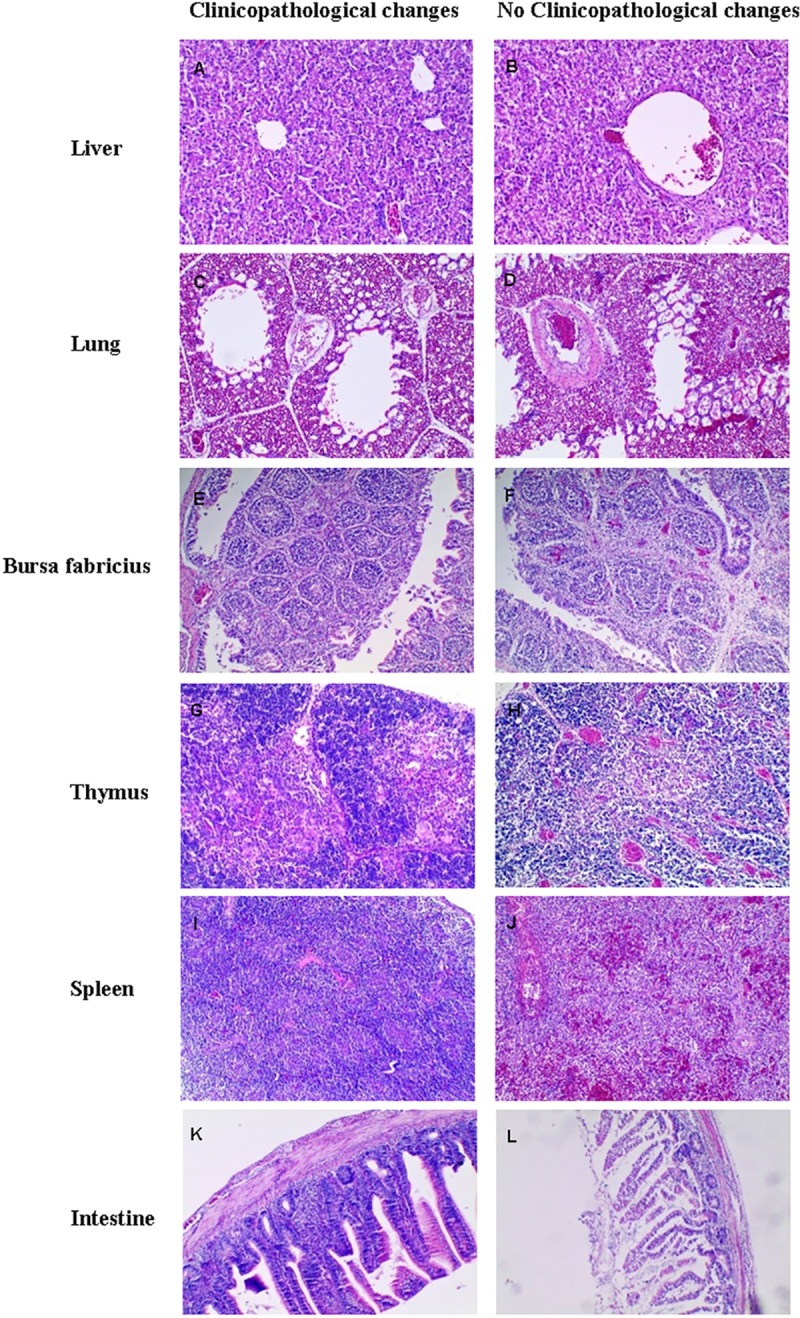
The histopathology changes of chicks. After acute dead period, the histopathology changes of the dead and euthanized chicks which observed clinicopathological changes from all groups showed the similar results. A. Liver from no clinicopathological changes chicks randomly. No lesion. B. Liver from presenting clinicopathological changes chick randomly. Cell swelling, steatosis, moderate to severe inflammatory cell infiltration were observed. C. Lung from no clinicopathological changes chicks randomly. No significant lesion. D. Lung from presenting clinicopathological changes chick randomly. Lymphoid infiltration, cellulose abounding in the alveolar cavity and slightly congestion. E. Bursa fabricius from no clinicopathological changes chicks randomly. No significant lesion. F. Bursa fabricius from presenting clinicopathological changes chick randomly. Unclear the tissue boundary, exfoliation of mucosal epithelium and interstitial connective tissue proliferation. G. Thymus from no clinicopathological changes chicks randomly. No significant lesion. H. Thymus from presenting clinicopathological changes chick randomly. Unclear the tissue boundary, moderate and severe congestion I. Spleen from no clinicopathological changes chicks randomly. No significant lesion. J. Spleen from symptomatic chick randomly. Unclear the tissue boundary, mild and moderate congestion, a large number of blood red cells were found in splenic sinusoid. K. Small intestine from no clinicopathological changes chicks randomly. No significant lesion. L. Small intestine from presenting clinicopathological changes chick randomly. Intestinal mucosal edema and mild and moderate intestinal villi shedding.

Firstly, the chicks died in day-1 post-infection showed some significant clinicopathological but no histopathological changes. After the prognosis period, there was no lesions of chicks in blank group and all organs were observed as normal according to their size, shape and consistency. After acute death period, the histopathology changes of dead chicks and the euthanized chicks from all groups showed the similar results that there were no significant histopathology findings in the chicks, which did not show clinicopathological changes (Fig 4).

From all groups except blank group, there were histopathological changes including moderate to severe lymphoid infiltration, swelling and steatosis in liver. Furthermore, histopathology changes of the lung included mild and moderate lymphoid infiltration, cellulose abounding in the alveolar cavity and slightly congestion in all above groups. In addition, there were unclear boundary of spleen, bursa fabricius and thymus, and the exfoliation of mucosal epithelium and interstitial connective tissue proliferation of bursa fabricius were observed in the part of these groups. There were significant change with moderate congestion in thymus and spleen. In the histopathological changes of small intestine, there were mainly changes including intestinal mucosal edema and mild and moderate intestinal villi shedding. The histopathology findings were consistent with clinicopathological changes.

### Relative expression of inflammatory Cytokine

**Fig 5:**
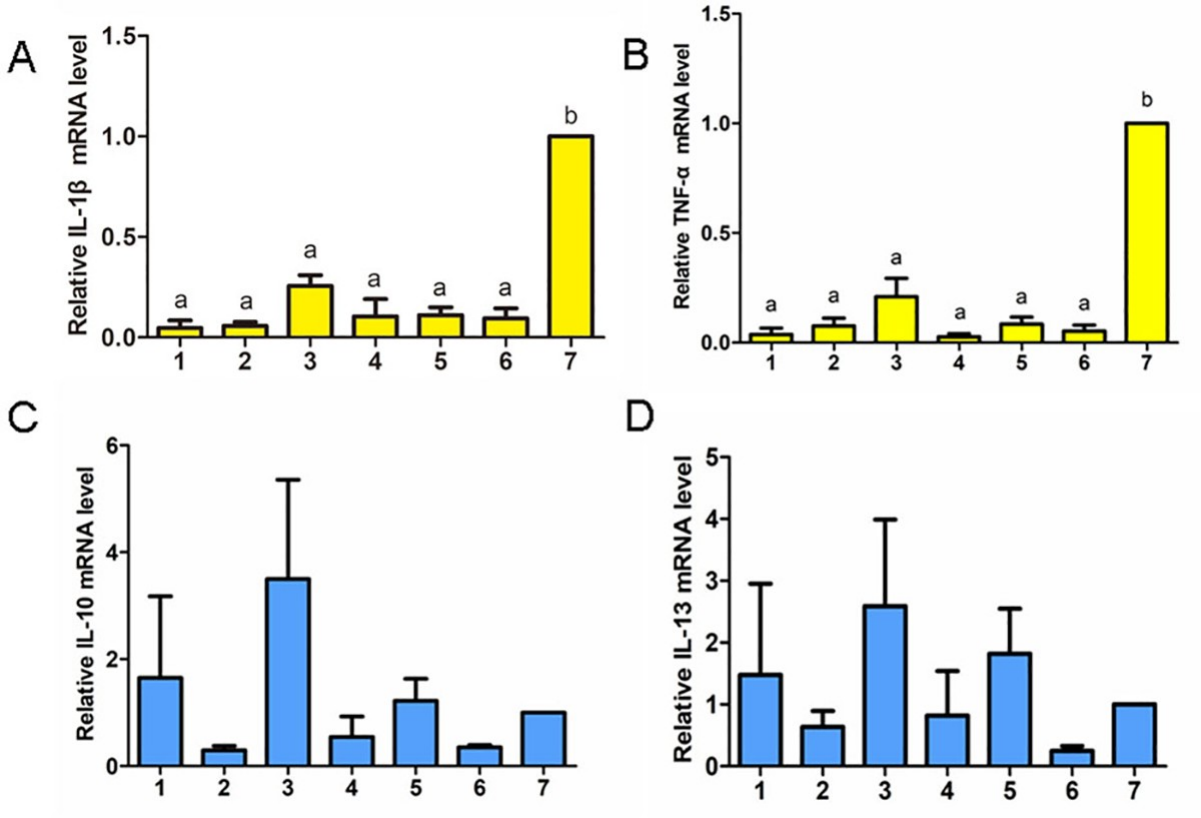
Pro-inflammatory cytokines and anti-inflammatory cytokines mRNA levels of chicks from all groups after the prognosis period. The pro-inflammatory cytokines are shown in yellow color, and the anti-inflammatory cytokines are shown in blue color. The groups with different letters are significantly different (P < 0.05).

The liver mRNA expression of IL-1β and TNF-α were illustrated in Fig 5A & 5B, respectively. Compared with blank group, there was no significant changes in the IL-1β and TNF-α mRNA expression levels after prognosis in the treatment groups. Notably, there were significantly lower IL-1β and TNF-α mRNA expression in treatment groups than blank group (P<0.05).

The liver mRNA expression of IL-10 and IL-13 were illustrated in Fig 5C & 5D, respectively. There were no significant differences in IL-10 and IL-13 mRNA expression after prognosis in any treatment groups compared with the blank group (P>0.05).

## Discussion

In this study, in order to assess the synergy functions of surfactin and amoxicillin on APEC infection, we evaluated the results of mortality, histopathology, liver bacterial loads and the relative expression of inflammatory cytokines. The mortality confirmed that surfactin can help amoxicillin to handle the APEC infection. In the meantime, as prognosis evaluating indices, histopathology, liver bacterial load and relative expression of inflammatory cytokine could furtherly confirm the effects of surfactin and amoxicillin. According to the results of these assays, the proportion of animals with good prognosis of each group from group 1 to 7 was 13%, 0, 33%, 0, 20%, 0 and 100%, respectively.

In this study, there was a significant difference between susceptibility tests *in vitro* and animal trials on treatment of APEC infection. The activity of surfactin as enhancer had been confirmed. First of all, surfactant component could decrease the inflammatory affect and lessen the histopathological changes. Compared with blank group, the pro-inflammatory cytokine IL-1β and TNF-α expression levels from other groups were similar, the results demonstrate that the infection of APEC changed from acute period to prognosis. The anti-inflammatory cytokine IL-10 and IL-13 expression levels in all treated groups showed no significant difference from the blank group, indicating that the infection may be controlled. In addition, compared the lesion score and histopathological changes with positive control and other groups, lesion scores in group 3 and 5 were lower than others, and presented relatively mild histopathological changes. All results demonstrated that surfactin have ability to lessen the damage of the chicks significantly as the function of helper for the property of surfactant proteins that agglutinating the bacterial[18], preventing bacterial dissemination, enhancing the pathogen uptake by phagocyte [18] and modifying immune response[19, 20].

Secondly, the amoxicillin belongs to β-lactams antibiotics which could destroy bacterial cell wall, and the surfactant component could increase the permeability of the cell membrane[21], the combination of amoxicillin and surfactin could facilitate the approach of amoxicillin to cell wall.

Thirdly, surfactant component was synthesized by alveolar type cell II[22] and had many properties as the above described. Furthermore, lung is the first colony site during the APEC infection progress in nature and the bacterial could down- regulate the expression of surfactant proteins during the pathogen infection[23–25]. As studies shown that mice deficient in surfactant proteins exhibit impaired clearance against various pathogen infections, including *group B Streptococcus*[26, 27], *Pseudomonas aeruginosa* [28], and *respiratory syncytial virus* [29]. Therefore, the supplement of surfactant component against APEC infection is potential effective treatment.

The amount of *E*.*coli* in the air of broiler house is about 10^5^−10^6^/g [30], and the research of Jeffrey suggested that the prevalence of pathogenic *E*. *coli* in the broiler house was independent of the prevalence of other commensal or environmental *E*. *coli* [31]. Compared with that the amount, there was 3x10^8^ CFU/ml directly infected issue as a serve experiment condition in this study. According to the results, surfactin as an enhancer could increase the survival rate under the huge amount APEC attack. Hence, the treatment may be performed better in regular infection progress of APEC.

In summary, we presented the novel antimicrobial adjuvant that effectively worked in combination with existing antimicrobial therapies against AEPC both in *vitro* and in *vivo*. Further studies are needed to uncover the molecular mechanisms of antibiotic sensitization induced by surfactin, and better develop surfactin as an antibiotic resistance adjuvant agent for other infection.

## Supporting information

S1 Table Score of pathology lesion following dead chicks and euthanized chicks after acute dead period. (DOCX)

S2 Table Bacterial load of dead chicks and euthanized chicks’ liver during the experiment. (DOCX)

S3 Table Pro-inflammatory cytokines IL-1β mRNA levels of chicks from all groups after the prognosis period. (DOCX)

S4 Table Pro-inflammatory cytokines TNF-α mRNA levels of chicks from all groups after the prognosis period. (DOCX)

S5 Table Anti-inflammatory cytokines IL-10 mRNA levels of chicks from all groups after the prognosis period. (DOCX)

S6 Table Anti-inflammatory cytokines IL-13 mRNA levels of chicks from all groups after the prognosis period. (DOCX)

## References

[1] Nhung NT, Chansiripornchai N, Carrique-Mas JJ. Antimicrobial Resistance in Bacterial Poultry Pathogens: A Review. Frontiers in veterinary science. 2017;4:126 Epub 2017/08/30. 10.3389/fvets.2017.00126 28848739 PMC5554362

[2] Marshall BM LS. Food animals and antimicrobials: impacts on human health. CLIN Microbiol Rev. 2011;24:718–33. 10.1128/CMR.00002-11 21976606 PMC3194830

[3] Lutful Kabir SM. Avian colibacillosis and salmonellosis: a closer look at epidemiology, pathogenesis, diagnosis, control and public health concerns. International journal of environmental research and public health. 2010;7(1):89–114. Epub 2010/03/03. 10.3390/ijerph7010089 20195435 PMC2819778

[4] Abu-Basha EA. In vitro susceptibility of resistant Escherichia coli field isolates to antimicrobial combinations. The Journal of Applied Poultry Research. 2012;21(3):595–602. 10.3382/japr.2011-00500

[5] Braga JFV, Chanteloup NK, Trotereau A, Baucheron S, Guabiraba R, Ecco R, et al Diversity of Escherichia coli strains involved in vertebral osteomyelitis and arthritis in broilers in Brazil. BMC veterinary research. 2016;12(1):140 Epub 2016/07/16. 10.1186/s12917-016-0762-0 27417195 PMC5477814

[6] Chansiripornchai N MS, Boonkhum P. Antimicrobial sensitivity of avian pathogenic Escherichia coli (APEC) isolated from chickens during 2007–2010. Thai J Vet Med. 2011;(41):519–22. 10.1186/1751-0147-53-64 22136406 PMC3247828

[7] Marks LR, Clementi EA, Hakansson AP. Sensitization of Staphylococcus aureus to methicillin and other antibiotics in vitro and in vivo in the presence of HAMLET. PloS one. 2013;8(5):e63158 Epub 2013/05/08. 10.1371/journal.pone.0063158 23650551 PMC3641093

[8] Fadli M, Chevalier J, Saad A, Mezrioui NE, Hassani L, Pages JM. Essential oils from Moroccan plants as potential chemosensitisers restoring antibiotic activity in resistant Gram-negative bacteria. International journal of antimicrobial agents. 2011;38(4):325–30. Epub 2011/07/15. 10.1016/j.ijantimicag.2011.05.005 . 21752605

[9] Yeh MS, Wei YH, Chang JS. Enhanced production of surfactin from Bacillus subtilis by addition of solid carriers. Biotechnology progress. 2005;21(4):1329–34. Epub 2005/08/06. 10.1021/bp050040c . 16080719

[10] Wu YS, Ngai SC, Goh BH, Chan KG, Lee LH, Chuah LH. Anticancer Activities of Surfactin and Potential Application of Nanotechnology Assisted Surfactin Delivery. Front Pharmacol. 2017;8:761 Epub 2017/11/11. 10.3389/fphar.2017.00761 29123482 PMC5662584

[11] Chen WC JR, Wei YH. Applications of a lipopeptide biosurfactant, surfactin, produced by microorganisms. Biochemical Engineering Journal. 2015;103:158–69.

[12] Mates SM, Patel L, Kaback HR, Miller MH. Membrane potential in anaerobically growing Staphylococcus aureus and its relationship to gentamicin uptake. Antimicrobial agents and chemotherapy. 1983;23(4):526–30. Epub 1983/04/01. 10.1128/aac.23.4.526 6859831 PMC184693

[13] Shahverdi AR, Fakhimi A, Zarrini G, Dehghan G, Iranshahi M. Galbanic acid from Ferula szowitsiana enhanced the antibacterial activity of penicillin G and cephalexin against Staphylococcus aureus. Biological & pharmaceutical bulletin. 2007;30(9):1805–7. Epub 2007/09/11. 10.1248/bpb.30.1805 . 17827745

[14] Braga LC, Leite AA, Xavier KG, Takahashi JA, Bemquerer MP, Chartone-Souza E, et al Synergic interaction between pomegranate extract and antibiotics against Staphylococcus aureus. Canadian journal of microbiology. 2005;51(7):541–7. Epub 2005/09/22. 10.1139/w05-022 . 16175202

[15] Goren E. Observations on experimental infection of chicks with Escherichia coli. Avian pathology: journal of the WVPA. 1978;7(2):213–24. Epub 1978/04/01. 10.1080/03079457808418274 . 18770374

[16] Alber A, Costa T, Chintoan-Uta C, Bryson KJ, Kaiser P, Stevens MP, et al Dose-dependent differential resistance of inbred chicken lines to avian pathogenic Escherichia coli challenge. Avian pathology: journal of the WVPA. 2018:1–33. Epub 2018/12/21. 10.1080/03079457.2018.1562154 . 30570345

[17] Bancroft JD, Cook H. C. Manual of histological techniques and their diagnostic applications. Churchill Livingstone London 1999.

[18] Nayak A, Dodagatta-Marri E, Tsolaki AG, Kishore U. An Insight into the Diverse Roles of Surfactant Proteins, SP-A and SP-D in Innate and Adaptive Immunity. Frontiers in immunology. 2012;3:131 Epub 2012/06/16. 10.3389/fimmu.2012.00131 22701116 PMC3369187

[19] Brinker KG, Garner H, Wright JR. Surfactant protein A modulates the differentiation of murine bone marrow-derived dendritic cells. American journal of physiology Lung cellular and molecular physiology. 2003;284(1):L232–41. Epub 2002/10/22. 10.1152/ajplung.00187.2002 . 12388334

[20] Cheng G, Ueda T, Nakajima H, Nakajima A, Kinjyo S, Motojima S, et al Suppressive effects of SP-A on ionomycin-induced IL-8 production and release by eosinophils. International archives of allergy and immunology. 1998;117 Suppl 1:59–62. Epub 1998/10/06. 10.1159/000053574 . 9758900

[21] Wu H, Kuzmenko A, Wan S, Schaffer L, Weiss A, Fisher JH, et al Surfactant proteins A and D inhibit the growth of Gram-negative bacteria by increasing membrane permeability. The Journal of clinical investigation. 2003;111(10):1589–602. Epub 2003/05/17. 10.1172/JCI16889 12750409 PMC155045

[22] Han S, Mallampalli RK. The Role of Surfactant in Lung Disease and Host Defense against Pulmonary Infections. Annals of the American Thoracic Society. 2015;12(5):765–74. Epub 2015/03/06. 10.1513/AnnalsATS.201411-507FR 25742123 PMC4418337

[23] Miakotina OL, McCoy DM, Shi L, Look DC, Mallampalli RK. Human adenovirus modulates surfactant phospholipid trafficking. Traffic. 2007;8(12):1765–77. Epub 2007/09/28. 10.1111/j.1600-0854.2007.00641.x . 17897321

[24] Haczku A, Atochina EN, Tomer Y, Chen H, Scanlon ST, Russo S, et al Aspergillus fumigatus-induced allergic airway inflammation alters surfactant homeostasis and lung function in BALB/c mice. American journal of respiratory cell and molecular biology. 2001;25(1):45–50. Epub 2001/07/27. 10.1165/ajrcmb.25.1.4391 . 11472974

[25] Bruce SR, Atkins CL, Colasurdo GN, Alcorn JL. Respiratory syncytial virus infection alters surfactant protein A expression in human pulmonary epithelial cells by reducing translation efficiency. American journal of physiology Lung cellular and molecular physiology. 2009;297(4):L559–67. Epub 2009/06/16. 10.1152/ajplung.90507.2008 19525387 PMC2770795

[26] LeVine AM, Bruno MD, Huelsman KM, Ross GF, Whitsett JA, Korfhagen TR. Surfactant protein A-deficient mice are susceptible to group B streptococcal infection. J Immunol. 1997;158(9):4336–40. Epub 1997/05/01. 10.1165/ajrcmb.19.4.3254 . 9126996

[27] LeVine AM, Kurak KE, Wright JR, Watford WT, Bruno MD, Ross GF, et al Surfactant protein-A binds group B streptococcus enhancing phagocytosis and clearance from lungs of surfactant protein-A-deficient mice. American journal of respiratory cell and molecular biology. 1999;20(2):279–86. Epub 1999/01/28. 10.1165/ajrcmb.20.2.3303 . 9922219

[28] LeVine AM, Kurak KE, Bruno MD, Stark JM, Whitsett JA, Korfhagen TR. Surfactant protein-A-deficient mice are susceptible to Pseudomonas aeruginosa infection. American journal of respiratory cell and molecular biology. 1998;19(4):700–8. Epub 1998/10/08. 10.1165/ajrcmb.19.4.3254 . 9761768

[29] LeVine AM, Gwozdz J, Stark J, Bruno M, Whitsett J, Korfhagen T. Surfactant protein-A enhances respiratory syncytial virus clearance in vivo. The Journal of clinical investigation. 1999;103(7):1015–21. Epub 1999/04/09. 10.1172/JCI5849 10194474 PMC408263

[30] Saif YM. DISEASES OF POULTRY, 10th edn Blackwell, London 2011.

[31] Jeffrey JS, Singer RS, O'Connor R, Atwill ER. Prevalence of pathogenic Escherichia coli in the broiler house environment. Avian diseases. 2004;48(1):189–95. Epub 2004/04/14. 10.1637/7043 . 15077814

