# Supplementary material for: Sensitization of avian pathogenic Escherichia coli to amoxicillin in vitro and in vivo in the presence of surfactin
Source: PLoS One. 2019 Sep 12;14(9):e0222413. doi: 10.1371/journal.pone.0222413 (PMC6742356; doi:10.1371/journal.pone.0222413)
Supplement: S1 Table — (DOCX) [file pone.0222413.s001.docx]

**S1 Table. Score of pathology lesion following dead chicks and euthanized chicks after acute dead period.**

| group | 1 | 2 | 3 | 4 | 5 | 6 | 7 |
| --- | --- | --- | --- | --- | --- | --- | --- |
| pathology lesion score |  |  |  |  |  |  | 0 |
|  |  |  |  |  |  |  | 0 |
|  |  |  |  |  |  |  | 0 |
|  |  |  |  |  |  |  | 0 |
|  |  |  |  | 4 |  |  | 0 |
|  |  |  | 4 | 6 | 4 |  | 0 |
|  |  | 2 | 0 | 3 | 6 |  | 0 |
|  | 5 | 4 | 0 | 5 | 4 | 3 | 0 |
|  | 5 | 4 | 5 | 5 | 4 | 4 | 0 |
|  | 5 | 5 | 1 | 5 | 5 | 5 | 0 |
|  | 6 | 5 | 0 | 4 | 4 | 4 | 0 |
|  | 5 | 5 | 4 | 5 | 0 | 6 | 0 |
|  | 6 | 6 | 1 | 4 | 1 | 6 | 0 |
|  | 1 | 5 | 0 | 3 | 0 | 4 | 0 |
|  | 1 | 5 | 1 | 3 | 1 | 0 | 0 |
