# Supplementary material for: Sensitization of avian pathogenic Escherichia coli to amoxicillin in vitro and in vivo in the presence of surfactin
Source: PLoS One. 2019 Sep 12;14(9):e0222413. doi: 10.1371/journal.pone.0222413 (PMC6742356; doi:10.1371/journal.pone.0222413)
Supplement: S2 Table — (DOCX) [file pone.0222413.s002.docx]

**S2 Table. Bacterial load of dead chicks and euthanized chicks’ liver during the experiment.**

| group | 1 | 2 | 3 | 4 | 5 | 6 | 7 |
| --- | --- | --- | --- | --- | --- | --- | --- |
| bacterial load (10^5 CFU/ml) | 70.5 | 67 | 8.5 | 104 | 159 | 170.5 | 0 |
|  | 52 | 66.5 | 35 | 120 | 191 | 138.7 | 0 |
|  | 127.5 | 164 | 17.1 | 4.5 | 156 | 116.7 | 0 |
|  | 131.5 | 66 | 0 | 19 | 55 | 89 | 0 |
|  | 17.5 | 33 | 0.05 | 24.5 | 9.5 | 152.5 | 0 |
|  | 6.5 | 68 | 8.7 | 92 | 124 | 120 | 0 |
|  | 8.05 | 49 | 0 | 65 | 48 | 68 | 0 |
|  | 46.5 | 13.5 | 0 | 45 | 119 | 40.5 | 0 |
|  | 25.3 | 31.5 | 1.36 | 85 | 61 | 16 | 0 |
|  | 10.55 | 4.5 | 0 | 74 | 41 | 78.6 | 0 |
|  | 15.5 | 0.9 | 0 | 1 | 0.68 | 11 | 0 |
|  | 58 | 0 | 0 | 1 | 0 | 4.8 | 0 |
|  | 3.9 | 2.22 | 0 | 5 | 0 | 13.7 | 0 |
|  | 0 | 0.206 | 0 | 0 | 0 | 2.6 | 0 |
|  | 0 | 0.135 | 0 | 0 | 0 | 0.198 | 0 |
