# Supplementary material for: Sensitization of avian pathogenic Escherichia coli to amoxicillin in vitro and in vivo in the presence of surfactin
Source: PLoS One. 2019 Sep 12;14(9):e0222413. doi: 10.1371/journal.pone.0222413 (PMC6742356; doi:10.1371/journal.pone.0222413)
Supplement: S3 Table — (DOCX) [file pone.0222413.s003.docx]

**S3 Table. Pro-inflammatory cytokines IL-1β mRNA levels of chicks from all groups after the prognosis period.**

| group | 1 | 2 | 3 | 4 | 5 | 6 | 7 |
| --- | --- | --- | --- | --- | --- | --- | --- |
| IL-1β relative expression level | 0.373874 | 0.146396 | 0.121622 | 0.087838 | 0.191441 | 0.362613 | 1 |
|  | 0.096847 | 0.103604 | 0.132883 | 0.876126 | 0.265766 | 0.353604 | 1 |
|  |  | 0.096847 | 0.617117 | 0.036036 | 0.220721 | 0.22973 | 1 |
|  |  | 0.096847 | 0.385135 | 0.038288 | 0.306306 |  | 1 |
|  |  | 0.128378 | 0.054054 | 0.009009 | 0.117117 |  | 1 |
|  |  |  | 0.281532 |  |  |  |  |
|  |  |  | 0.274775 |  |  |  |  |
|  |  |  | 0.087838 |  |  |  |  |
|  |  |  | 0.218469 |  |  |  |  |
|  |  |  | 0.380631 |  |  |  |  |
|  |  |  |  |  |  |  |  |
|  |  |  |  |  |  |  |  |
|  |  |  |  |  |  |  |  |
|  |  |  |  |  |  |  |  |
|  |  |  |  |  |  |  |  |
