# Supplementary material for: Sensitization of avian pathogenic Escherichia coli to amoxicillin in vitro and in vivo in the presence of surfactin
Source: PLoS One. 2019 Sep 12;14(9):e0222413. doi: 10.1371/journal.pone.0222413 (PMC6742356; doi:10.1371/journal.pone.0222413)
Supplement: S4 Table — (DOCX) [file pone.0222413.s004.docx]

**S4 Table. Pro-inflammatory cytokines TNF-α mRNA levels of chicks from all groups after the prognosis period.**

| group | 1 | 2 | 3 | 4 | 5 | 6 | 7 |
| --- | --- | --- | --- | --- | --- | --- | --- |
| TNF-α relative expression level | 0.28067 | 0.327042 | 0.079727 | 0.032541 | 0.154572 | 0.152945 | 1 |
|  | 0.09437 | 0.069151 | 0.043117 | 0.143183 | 0.170029 | 0.202571 | 1 |
|  |  | 0.05288 | 0.929873 | 0.030101 | 0.166775 | 0.17247 | 1 |
|  |  | 0.071591 | 0.228604 | 0.052066 | 0.275789 |  | 1 |
|  |  | 0.235112 | 0.050439 | 0.009762 | 0.077286 |  | 1 |
|  |  |  | 0.09437 |  |  |  |  |
|  |  |  | 0.195249 |  |  |  |  |
|  |  |  | 0.062642 |  |  |  |  |
|  |  |  | 0.135047 |  |  |  |  |
|  |  |  | 0.272535 |  |  |  |  |
|  |  |  |  |  |  |  |  |
|  |  |  |  |  |  |  |  |
|  |  |  |  |  |  |  |  |
|  |  |  |  |  |  |  |  |
|  |  |  |  |  |  |  |  |
