# Supplementary material for: Sensitization of avian pathogenic Escherichia coli to amoxicillin in vitro and in vivo in the presence of surfactin
Source: PLoS One. 2019 Sep 12;14(9):e0222413. doi: 10.1371/journal.pone.0222413 (PMC6742356; doi:10.1371/journal.pone.0222413)
Supplement: S5 Table — (DOCX) [file pone.0222413.s005.docx]

**S5 Table. Anti-inflammatory cytokines IL-10 mRNA levels of chicks from all groups after the prognosis period.**

| group | 1 | 2 | 3 | 4 | 5 | 6 | 7 |
| --- | --- | --- | --- | --- | --- | --- | --- |
| IL-10 relative expression level | 3.174419 | 0.465116 | 0.372093 | 2.069767 | 1.127907 | 0.395349 | 1 |
|  | 0.127907 | 0.511628 | 0.395349 | 0.244186 | 0.046512 | 0.395349 | 1 |
|  |  | 0.162791 | 19.13953 | 0.197674 | 0.895349 | 0.255814 | 1 |
|  |  | 0.22093 | 2.27907 | 0.151163 | 1.430233 |  | 1 |
|  |  | 0.116279 | 0.151163 | 0.05814 | 2.593023 |  | 1 |
|  |  |  | 4.72093 |  |  |  |  |
|  |  |  | 1.593023 |  |  |  |  |
|  |  |  | 0.290698 |  |  |  |  |
|  |  |  | 6.034883 |  |  |  |  |
|  |  |  | 0.023256 |  |  |  |  |
|  |  |  |  |  |  |  |  |
|  |  |  |  |  |  |  |  |
|  |  |  |  |  |  |  |  |
|  |  |  |  |  |  |  |  |
|  |  |  |  |  |  |  |  |
