# Supplementary material for: Sensitization of avian pathogenic Escherichia coli to amoxicillin in vitro and in vivo in the presence of surfactin
Source: PLoS One. 2019 Sep 12;14(9):e0222413. doi: 10.1371/journal.pone.0222413 (PMC6742356; doi:10.1371/journal.pone.0222413)
Supplement: S6 Table — (DOCX) [file pone.0222413.s006.docx]

**S6 Table. Anti-inflammatory cytokines IL-13 mRNA levels of chicks from all groups after the prognosis period.**

| group | 1 | 2 | 3 | 4 | 5 | 6 | 7 |
| --- | --- | --- | --- | --- | --- | --- | --- |
| IL-13 relative expression level | 2.95082 | 0.491803 | 0.081967 | 3.688524 | 3.852459 | 0.409836 | 1 |
|  | 0 | 1.393443 | 0.327869 | 0 | 0.163934 | 0.163934 | 1 |
|  |  | 0.409836 | 13.77049 | 0.245902 | 0.163934 | 0.163934 | 1 |
|  |  | 0.245902 | 0.819672 | 0.081967 | 2.213115 |  | 1 |
|  |  |  | 0 | 0.081967 | 2.704918 |  |  |
|  |  |  | 2.213115 |  |  |  |  |
|  |  |  | 0.245902 |  |  |  |  |
|  |  |  | 1.721311 |  |  |  |  |
|  |  |  | 6.721312 |  |  |  |  |
|  |  |  | 0 |  |  |  |  |
|  |  |  |  |  |  |  |  |
|  |  |  |  |  |  |  |  |
|  |  |  |  |  |  |  |  |
|  |  |  |  |  |  |  |  |
|  |  |  |  |  |  |  |  |
